# The *gpsX *gene encoding a glycosyltransferase is important for polysaccharide production and required for full virulence in *Xanthomonas citri *subsp. *citri*

**DOI:** 10.1186/1471-2180-12-31

**Published:** 2012-03-09

**Authors:** Jinyun Li, Nian Wang

**Affiliations:** 1 Citrus Research and Education Center, Department of Microbiology and Cell Science, University of Florida, IFAS, 700 Experiment Station Road, Lake Alfred FL 33850, USA; 2 Department of Plant Pathology, China Agricultural University, Beijing 100193, China

## Abstract

**Background:**

The Gram-negative bacterium *Xanthomonas citri *subsp. *citri *(*Xac*) causes citrus canker, one of the most destructive diseases of citrus worldwide. In our previous work, a transposon mutant of *Xac *strain 306 with an insertion in the *XAC3110 *locus was isolated in a screening that aimed at identifying genes related to biofilm formation. The *XAC3110 *locus was named as *bdp24 *for biofilm-defective phenotype and the mutant was observed to be affected in extracellular polysaccharide (EPS) and lipopolysaccharide (LPS) biosynthesis and cell motility. In this study, we further characterized the *bdp24 *(XAC3110) gene (designated as *gpsX*) using genetic complementation assays and expanded the knowledge about the function of the *gpsX *gene in *Xac *pathogenesis by investigating the roles of *gpsX *in EPS and LPS production, cell motility, biofilm formation on host leaves, stress tolerance, growth *in planta*, and host virulence of the citrus canker bacterium.

**Results:**

The *gpsX *gene encodes a putative glycosyltransferase, which is highly conserved in the sequenced strains of *Xanthomonas*. Mutation of *gpsX *resulted in a significant reduction of the amount of EPS and loss of two LPS bands visualized on sodium dodecylsulphate- polyacrylamide gels. Biofilm assays revealed that the *gpsX *mutation affected biofilm formation by *Xac *on abiotic and biotic surfaces. The *gpsX *mutant showed delayed bacterial growth and caused reduced development of disease symptoms in susceptible citrus leaves. The *gpsX *mutant was more sensitive than the wild-type strain to various stresses, including the H_2_O_2 _oxidative stress. The mutant also showed attenuated ability in cell motility but not in flagellar formation. Quantitative reverse transcription-PCR assays indicated that mutation of *gpsX *did not affect the expression of virulence genes such as *pthA *in *Xac *strain 306. The affected phenotypes of the *gpsX *mutant could be complemented to wild-type levels by the intact *gpsX *gene.

**Conclusions:**

Taken together, our data confirm that the *gpsX *gene is involved in EPS and LPS synthesis and biofilm formation in *Xac *and suggest that the *gpsX *gene contributes to the adaptation of *Xac *to the host microenvironments at early stage of infection and thus is required for full virulence on host plants.

## Background

Citrus canker, caused by the Gram-negative plant pathogenic bacterium *Xanthomonas citri *subsp*. citri *(*Xac*) (syn. *Xanthomonas axonopodis *pv. *citri*) [1,2], is one of the most important diseases of citrus crop worldwide [3]. Citrus canker is widely distributed in wet subtropical citrus growing areas and affects most commercial citrus varieties [3,4]. The canker symptom is characterized by raised necrotic lesions on leaves, stems and fruit of infected trees; and in severe cases, defoliation, twig dieback, general tree decline, blemished fruit and premature fruit drop can occur [3,4]. Wind-blown rain is the primary short- to medium-distance spread mechanism for citrus canker and long-distance dissemination is usually caused by transportation of infected citrus fruits and plant materials [5]. The decrease of yield and less value or entirely unmarketable of infected fruit are responsible for serious economic losses [3]. Moreover, this disease has a significant impact on commerce due to restrictions to national and international fruit trade from canker-affected areas [3]. Economic losses are also resulting from costly eradication programs and heave use of chemical treatments such as copper-based bactericides for prevention from and control of citrus canker disease [6].

Several members of the genus *Xanthomonas*, including *Xac*, have been extensively studied as model organisms to dissect the mechanisms of bacterium-plant interactions, including the molecular basis of pathogenesis and plant disease resistance [7-9]. *Xac *is considered to be a hemibiotrophic pathogen because it is able to obtain nutrients from living host cells, multiply in the apoplast (intercellular spaces) and then infect neighbouring tissues, after invading citrus host directly through natural openings, such as stomata, and through wounds [4]. The apoplast is a nutrient-limited environment that is guarded by plant defenses [10]. *Xac*, like many other plant pathogenic bacteria, has evolved several strategies to adapt to and successfully colonize this *in planta *niche by overcoming the plant defense and creating a favourable environment for bacterial growth, which include, among others, the type III secretion system (TTSS) and its effectors, cell wall degrading enzymes, and bacterial polysaccharides [8]. Bacterial polysaccharides of plant pathogenic bacteria, including extracellular polysaccharides (EPS), lipopolysaccharides (LPS) and capsular polysaccharides (CPS), have been shown to play a role in a number of different diseases. They collectively or individually contribute to the bacterial growth and survival *in planta*, and also are involved in the bacterium-plant interaction [8].

Progress has been made in elucidating the biosynthesis of bacterial polysaccharides over the decades [11]. The biosynthesis of bacterial polysaccharides occurs in successive steps. Firstly, nucleotide sugars are produced, which provide specifically activated monosaccharides as precursors for the subsequent synthesis steps. Secondly, monosaccharide moieties from the nucleotide sugar precursors are sequentially transferred by highly specific glycosyltransferases (GTs) to sugar or nonsugar acceptors, resulting in the formation of saccharide repeating units. Finally, the repeating units are polymerized and the polymer is exported from the cell. Bacterial GTs have been reported to be involved not only in the biosynthesis of EPS, LPS, CPS, peptidoglycans, and glycolipids, but also in protein and lipid glycosylation, showing enormous diversity of biological functions and substrates [12-14].

Much effort has been made to identify genes that encode GTs, their enzymatic functions, and the structures of these enzymes. Currently, there are more than 94 GT families in the Carbohydrate-Active EnZymes (CAZy) database (http://www.cazy.org) based on amino acid sequence similarities [15,16]. Two main three-dimensional folds, named GT-A and GT-B, have been observed for structures of nucleotide sugar-dependent GTs [12,13]. There is high sequence variability, although the relatively low structural variety and it is not yet possible to reliably predict the precise function of a given GT.

Mutations in GTs encoding genes have profound biological effects in a variety of bacteria. For example, mutation in s*psA *of *Bacillus subtilis *resulted in an altered spore coat [17]. In *Escherichia coli *strain 2787 (O126:H27), an *aah*-deletion mutant was attenuated in adherence to host cells [18], and the *pgaC *mutant of *E. coli *K-12 was impaired in surface binding, intercellular adhesion, and biofilm formation [19]. Mutation of *orfN *in *Pseudomonas aeruginosa *PAK affected the flagellin glycosylation [20]. In *X. campestris *pv. *campestris *strain 8004, mutation of *xagB (XC_3555) *led to decreased EPS production, abolished biofilm formation and attenuated bacterial resistance to oxidative stress [21], and the *XC_3814 *mutant was significantly reduced both in EPS production and virulence on host plants [22]; while the *rfbC *mutation in *Xac *strain 306 resulted in altered O-antigen of LPS, reduced biofilm formation and attenuated bacterial resistance to environmental stresses [23].

In our previous work, an EZ-Tn5 transposon mutant of *Xac *strain 306 with an insertion in the *XAC3110 *locus was isolated in a screening that aimed at identifying genes involved in biofilm formation. The *XAC3110 *locus was named as *bdp24 *for biofilm-defective phenotype and the mutant was observed to be affected in EPS and LPS biosynthesis, cell motility and biofilm formation on abiotic surfaces [24]. Due to the nature of our previous study in genome-wide identification of biofilm related genes, we focused on big picture rather than individual genes. It is necessary to further characterize the novel genes identified in our previous study and provide conclusive genetic evidence in complementation. In this study, we further characterized the *bdp24 *(XAC3110) gene (renamed as *gpsX*) that encodes a putative glycosyltransferase using genetic complementation assays. The data obtained confirmed that the novel gene *gpsX *plays a role in EPS and LPS biosynthesis, cell motility, biofilm formation on abiotic surfaces and host leaves, stress tolerance, growth *in planta*, and host virulence of the citrus canker bacterium. These findings suggest that the *gpsX *gene contributes to the adaptation of *Xac *to the host microenvironments at early stage of infection and thus is required for full virulence on host plants.

## Results

### The *gpsX *gene encodes a glycosyltransferase involved in polysaccharide biosynthesis in *X. citri *subsp. *citri*

**Figure 1:**
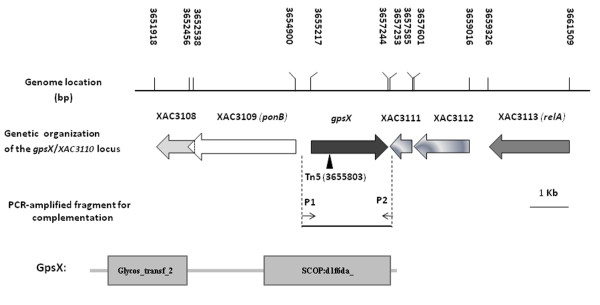
**Schematic diagram of the *gpsX *(XAC3110) gene in the genome of *X. citri *subsp. *citri *strain 306**. Open arrows indicate length, location and orientation of the ORFs. The triangle indicates the EZ-Tn*5 *insertion site in mutant 223 G4 (*gpsX-)*. Gene colour represents operon membership. The middle element shows the 2299 bp PCR fragment cloned into the plasmid pUFR053 for complementation of the *gpsX *mutant 223 G4 (*gpsX-)*. The lower element shows domain structure analyses of the putative GpsX protein. The domain structure prediction was performed using the SMART program program http://smart.embl-heidelberg.de/. Domain symbols: Glycos_transf_2: glycosyltransferase family 2 domain; SCOP:d1f6da_: UDP-Glycosyltransferase/glycogen phosphorylase superfamily.

**Figure 2:**
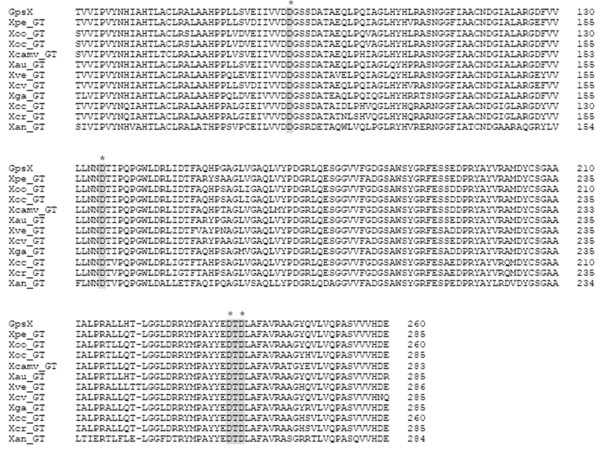
**Sequence alignment of N-terminal residues of GpsX including the Glycosyltransferase family 2 domain and its glycosyltransferase homologues**. The motifs predicted to be involved in the catalytic activity of GpsX are highlighted in gray background and indicated by the symbols (*). Abbreviations are as follows: GpsX, *X. citri *subsp. *citri *glycosyltransferase (NCBI Accession No. NP_643419); Xpe_GT, *X. perforans *glycosyltransferase (ZP_08188792); Xoo_GT, *X. oryzae *pv. *oryzae *glycosyltransferase (YP_200377); Xoc_GT, *X. oryzae *pv*. oryzicola *glycosyltransferase (ZP_02244158); Xcamv_GT, *X. campestris *pv. *vasculorum *glycosyltransferase (ZP_06483586); Xau_GT, *X. fuscans *subsp. *aurantifolii *glycosyltransferase (ZP_06732262); Xve_GT, *X. vesicatoria *glycosyltransferase (ZP_08176519); Xcv_GT, *X. campestris *pv. *vesicatoria *glycosyltransferase (YP_364973); Xga_GT, *X. gardneri *glycosyltransferase (ZP_08185487); Xcc_GT, *X. campestris *pv. *campestris *glycosyltransferase (YP_242265); Xcr_GT, *X. campestris *pv. *raphani *glycosyltransferase (AEL08167); Xan_GT, *X. albilineans *glycosyltransferase (YP_003376724).

The *XAC3110 *locus was identified as a biofilm formation-related gene of *bdp24 *that may be involved in EPS and LPS biosynthesis, following screening a transposon insertion mutant library of *Xac *strain 306 in our earlier work [24]. The *XAC3110 *open reading frame (ORF) is 2028 bp in length and located in the genome sequence at position 3655217-3657244 (Figure 1). *XAC3110 *consists of a single transcriptional unit, whereas the adjacent upstream and downstream genes were transcribed separately from this ORF in reverse orientation [25]. *XAC3110 *was annotated as a 675 aa glycosyltransferase [7]. The predicted pI and molecular weight (MW) of the putative enzyme are 6.67 and 73.9 kD (http://web.expasy.org/compute_pi/), respectively. The predicted protein contained a glycosyltransferase family 2 domain (PF00535, 2.00e-28) (residues 50-216) at the N-terminal and a UDP-Glycosyltransferase/glycogen phosphorylase superfamily domain (SCOP:d1f6da_, 2.00e-12) (residues 340-660) at the C-terminal (Figure 1). In addition, PSI-BLAST analysis revealed that the XAC3110 belongs to glycosyltransferase family II (GT-2) in the Pfam Protein Family Database [26]. The predicted XAC3110 protein processes several conserved catalytic residues of glycosyltransferases including the DXD motif (D^234^TD^236^) for the divalent metal ion binding in glycosyltransferases with a common GT-A structural fold [27,28] (Figure 2). Database search revealed that XAC3110 are highly conserved in other sequenced *Xanthomonas *species, including *X. oryzae*, *X. campestris, X. fuscans, X. perforans*, *X. vesicatoria, X. gardneri*, and *X. albilineans*, with over 70% amino acid identity (Table 1). All these homologues are putative glycosyltransferases with unknown functions. Their homologues with about 35-70% identity are also present in *Acetobacter aceti*, *Clostridium *spp., *Xylella fastidiosa*, *Chlorobium phaeobacteroides*, *Saccharopolyspora erythraea*, *Thiorhodococcus drewsii*, *Rhodospirillum centenum*, *Stenotrophomonas *spp., and *Burkholderia *spp.; among which, several are putative GT-2 proteins (data not shown). These findings strongly suggested that XAC3110 may be a member of GT-2. Collectively, given the role in polysaccharide production (see below), the *bdp24 *(XAC3110) gene was renamed as *gpsX *(glycosyltransferase for polysaccharide synthesis in *X. citri *subsp. *citri*).

**Table 1:** GpsX/XAC3110 homologues in *Xanthomonas *spp

Strains *^a^*		Homologue			
	
	Gene/locus_tag	Putative product	Size (aa)	Domain structure *^b^*	Identity (%) *^c^*
*Xac *306	*gpsX/XAC3110*	glycosyltransferase	675	Glycos_transf_2 (1); SCOP:d1f6da_(1)	
*Xpe *91-118	*XPE_2818*	glycosyltransferase	700	Glycos_transf_2 (1); SCOP:d1f6da**_**(1)	97
*Xoo *KACC10331	*XOO1738*	glycosyltransferase	675	Glycos_transf_2 (1); Glycos_transf_1(1);	94
*Xoo *MAFF311018	*XOO_1639*	glycosyltransferase	700	Glycos_transf_2 (1);	94
*Xoo *PXO99A	*PXO_01594*	glycosyltransferase	700	Glycos_transf_2 (1)	94
*Xoc *BLS256	*Xoryp_010100016275*	glycosyltransferase	700	Glycos_transf_2 (1); Glycos_transf_1(1);	94
*Xcv *NCPPB702	*XcampvN_010100002613*	glycosyltransferase	698	Glycos_transf_2 (1); Glycos_transf_1(1);	94
*Xau *ICPB10535	*XAUC_30140*	glycosyltransferase	694	Glycos_transf_2 (1); Glycos_transf_1(1);	93
*Xau *ICPB11122	*XAUB_29140*	glycosyltransferase	694	Glycos_transf_2 (1); SCOP:d1f6da**_**(1)	93
*Xve *ATCC35937	*XVE_0383*	glycosyltransferase	701	Glycos_transf_2 (1); SCOP:d1f6da**_**(1)	93
*Xcv *85-10	*XCV3242*	glycosyltransferase	694	Glycos_transf_2 (1); SCOP:d1f6da**_**(1)	92
*Xga *ATCC19865	*XGA_4540*	glycosyltransferase	700	Glycos_transf_2 (1); SCOP:d1f6da**_**(1)	92
*Xcc *8004	*XC_1175*	glycosyltransferase	675	Glycos_transf_2 (1); Glycos_transf_1(1);	90
*Xcc *ATCC33913	*XCC2933*	glycosyltransferase	700	Glycos_transf_2 (1); Glycos_transf_1(1);	89
*Xcc *B100	*xccb100_1219*	hypothetical protein	700	Glycos_transf_2 (1); SCOP:d1f6da**_**(1)	89
*Xcr *756C	*XCR_3304*	glycosyltransferase	700	Glycos_transf_2 (1); SCOP:d1f6da**_**(1)	89
*Xan *GPE PC73	*XALc_2250*	glycosyltransferase	698	Glycos_transf_2 (1); Glycos_transf_1(1);	70

*^a ^Xac *306: *X. axonopodis *pv. *citri *strain 306 (GenBank accession number: AE008923); *Xpe *91-118: *X. perforans *91-118 (AEQW00000000); *Xoo *KACC10331*: X. oryzae *pv. *oryzae *KACC10331 (AE0135983); *Xoo *MAFF311018: *X. oryzae *pv. *oryzae *MAFF311018 (AP008229); *Xoo *PXO99A: *X. oryzae *pv. *oryzae *PXO99A (CP000967); *Xoc *BLS256: *X. oryzae *pv*. oryzicola *BLS256 (AAQN00000000); *Xcv *NCPPB702: *X. campestris *pv. *vasculorum *NCPPB702 (ACHS00000000); *Xau *ICPB10535*: X. fuscans *subsp. *aurantifolii *ICPB10535 (ACPY00000000); *Xau *ICPB11122: *X. fuscans *subsp. *aurantifolii *ICPB11122 (ACPX00000000); *Xve *ATCC35937: *X. vesicatoria *ATCC35937 (AEQV00000000); *Xcv *85*-*10: *X. campestris *pv. *vesicatoria *85-10 (AM039952); *Xga *ATCC19865: *X. gardneri *ATCC19865 (AEQX00000000); *Xcc *8004: *X. campestris *pv. *campestris *8004 (CP0000509); *Xcc *ATCC33913: *X. campestris *pv. *campestris *ATCC 33913 (AE008922); *Xcc *B100: *X. campestris *pv. *campestris *B100 (AM920689); *Xcr *756 C: *X. campestris *pv. *raphani *756 C (CP002789); *Xan *GPE PC73: *X. albilineans *GPE PC73(FP565176).*^b ^*Domains were predicted by the SMART program http://smart.embl-heidelberg.de/. Domain symbol: Glycos_transf_2, glycosyltransferase family 2 domain; SCOP:d1f6da_: UDP-Glycosyltransferase/glycogen phosphorylase superfamily; Glycos_transf_1, glycosyltransferase family 1 domain. The total number of the domains was indicated in the bracket. *^c^*According to a BLASTP search.

**Figure 3:**
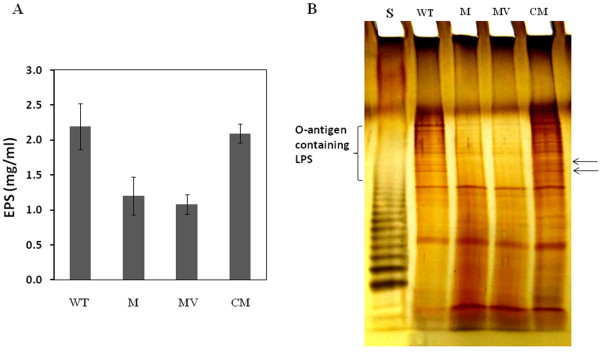
**GpsX is involved in polysaccharide biosynthesis in *X. citri *subsp. *citri***. (A) EPS production in NB medium supplemented with 2% (w/v) glucose by wild type strain 306 and its derivatives. The data presented are the means ± SD of triplicate measurements from a representative experiment; similar results were obtained in two other independent experiments. (B) Analysis of LPS synthesis. The LPSs produced by wild type strain 306 and its derivatives were extracted, subjected to SDS-PAGE analysis, and visualized by silver staining. The lost bands in the mutants are indicated by arrows. WT: wild-type strain 306; M: *gpsX *mutant C223 G4 (*gpsX-)*; MV: *gpsX *mutant 223G4V (*gpsX-) *with empty vector pUFR053; CM: complemented *gpsX *mutant C223 G4 (*gpsX+)*; S: LPS standard from *S*. *enterica *serovar Typhimurium (10 μg; Sigma). The experiments were repeated three times with similar results, and the results of only one experiment are presented.

To exclude the possibility of multiple EZ-Tn*5 *insertions in the genome of the *gpsX *mutant 223 G4 (*gpsX-) *(Table 2), complementation assays were performed for this mutant. The complementary plasmid pJU3110 with intact *gpsX *(Table 2) was transformed into the mutant 223 G4 (*gpsX-)*, and the complemented strain C223G4 (*gpsX+) *was assayed for EPS and LPS production. The results showed that the total EPS production of the *gpsX *mutant in NB containing 2% glucose at 24 hours post inoculation could be restored to the wild-type level by the plasmid pJU3110, but not by the empty vector pUFR053 (Figure 3A). Both the mutant strains 223 G4 (*gpsX-) *and 223G4V (*gpsX-) *produced significantly less EPS than the wild-type strain 306. The complemented strain C223G4 (*gpsX+) *had a similar level of EPS production to the wild-type strain. Sodium dodecylsulphate-polyacrylamide gel electrophoresis (SDS-PAGE) analysis showed that LPS of the *gpsX *mutant was different from that of the wild-type strain 306 (Figure 3B). Two bands corresponding to the O-antigen containing LPS were completely lost in the *gpsX *mutant, compared to wild type strain 306. The LPS pattern of the complemented *gpsX *mutant was similar with that of the wild-type strain 306. The empty vector pUFR053 did not complement LPS biosynthesis in the *gpsX *mutant (Figure 3B). These findings indicated that the transposon insertion mutation in *gpsX *could be complemented by the wild type ORF *in trans *and, the *gpsX *locus is involved in polysaccharides biosynthesis in *X. citri *subsp. *citri*.

**Table 2:** Bacterial strains and plasmidsa

Strains and plasmids	Characteristics	Reference or source
Strains		
*E. coli*DH5α	F^- ^*recA1 endA1 hsdR17 supE44 thi-1 gyrA96 relA1 *Δ (*argE-lacZYA*)*169 *φ80 *lazA *Δ M15	[29]
HB101	F^- ^*supE44, hsdS20(rB^- ^mB^-^), recA13, ara-14, proA2, lacY1, galK2, rpsL20, xyl-5, mtl-1, leuB6, thi*	[30]
*X. citri *subsp. *citri*		
306	Syn. *X*. *axonopodis *pv. *citri *strain 306; wild type, Rf^r^	[31]
223G4 (*gpsX-)*	*gpsX *(*XAC3110*):: EZ-Tn5 derivative of strain 306, Km^r^, Rf^r^	[24]
223G4V (*gpsX-)*	223G4 (*gpsX-*) containing pUFR053, Cm^r^, Gm^r^, Km^r^, Rf^r^	This study
C223G4 (*gpsX+)*	223G4 (*gpsX-*) containing pJU3110, Cm^r^, Gm^r^, Km^r^, Rf^r^	This study
Plasmids		
pRK2013	ColE1 Km^r ^Tra^+^, conjugation helper plasmid	[32]
pUFR053	IncW Mob^+^*mob*(P) *lac*Z^+ ^Par^+^, Cm^r^, Gm^r^, shuttle vector	[33]
pJU3110	2,299-bp *Kpn*I- *Hin*dIII fragment containing wild-type *gpsX *cloned in pUFR053; Cm^r^, Gm^r^	This study

*^a^*Ap^r^, Cm^r^, Gm^r^, Km^r^, and Rf^r ^indicate resistance to ampicillin, chloromycetin, gentamicin, kanamycin and rifamycin, respectively.

To further confirm the role of *gpsX *in polysaccharides biosynthesis, the EPS production of the mutants grown in XVM2 liquid medium supplemented with 2% of various carbohydrates was quantitatively estimated. As summarized in Table 3, the *gpsX *mutant produced about 30-50% less EPS than the wild-type strain 306 when cultured in fructose-, galactose-, glucose-, maltose-, mannose-, or sucrose-containing medium; while the EPS yield of the complemented mutant strains showed no significant difference from that of the wild-type. In contrast, no significant difference in capsule staining was observed between the *gpsX *mutant strain and the wild-type strain 306 in capsule assays (data not shown).

**Table 3:** EPS production in *X.citri *subsp. *citri *strainsa

Strain			EPS yield (g/L)				
	
	Fructose	Galactose	Glucose	Maltose	Mannose	Sucrose	Xylose
306	1.73 ± 0.23 a	1.08 ± 0.24 a	1.83 ± 0.17 a	1.22 ± 0. 11 a	1.54 ± 0.27 a	1.62 ± 0.18 a	1.38 ± 0. 21 a
223G4 (*gpsX-*)	0.83 ± 0.14 b	0.64 ± 0.11 b	1.22 ± 0.25 b	0.75 ± 0. 19 b	0.94 ± 0.12 b	0.68 ± 0.11 b	1.15 ± 0. 17 a
C223G4 (*gpsX+*)	1.91 ± 0.36 a	1.22 ± 0.25 a	1.96 ± 0.34 a	1.14 ± 0. 16 a	1.45 ± 0.19 a	1.76 ± 0.31 a	1.53 ± 0. 25 a

*^a ^*Strains were cultured in XVM2 liquid medium supplemented with various carbon sources. Data presented are means and standard errors of three replicates from one representative experiment and similar results were obtained in two other independent experiments. Different letters in each data column indicate significant differences at *P *< 0.05 (Student's t-test).

### GpsX was required for full virulence and growth of *X. citri *subsp. *citri *in host plants

**Figure 4:**
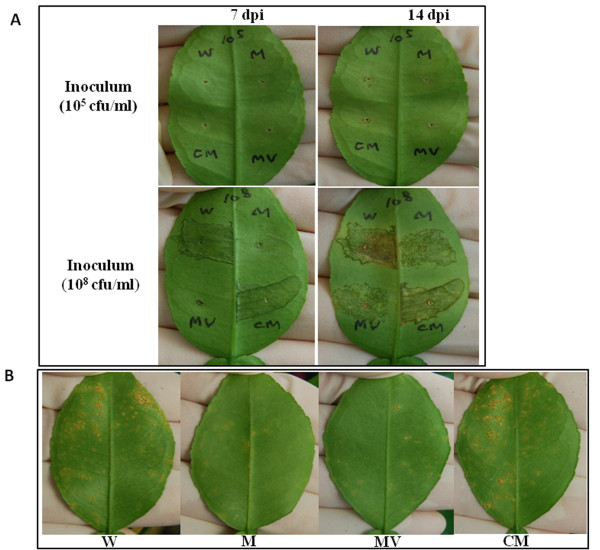
**GpsX is important for host virulence of *X. citri *subsp. *citri***. (A) Suspensions of each strain [approximately 10^5 ^and 10^8 ^cfu/ml, respectively] were inoculated into the intercellular spaces of fully expanded, immature grapefruit (*C*. *paradise *cv. Duncan) leaves by pressure infiltration with a needleless syringe. A representative leaf from four replicates was photographed at 7 and 14 dpi, respectively. W: wild-type strain 306; M: *gpsX *mutant 223 G4 (*gpsX-*); MV: *gpsX *mutant 223G4V (*gpsX-*) with empty vector pUFR053; CM: complemented *gpsX *mutant C223G4 (*gpsX+*). (B) Bacterial cell suspensions (approximately 10^8 ^cfu/ml) of wild-type strain 306 and its derivatives were inoculated onto fully expanded, immature grapefruit by spray. Images are representative of five independent replicates at 21 dpi.

Since both EPS and LPS have been demonstrated to contribute to host virulence of *X. citri *subsp. *citri *[23,34,35], we were interested in determining whether the *gpsX *gene is associated with pathogenicity of the canker bacterium. The virulence of the *gpsX *mutant was assessed on the host plant grapefruit using two inoculation methods: pressure infiltration and spray. Upon infiltration at a concentration 10^5 ^colony-forming units (cfu)/ml, no disease symptom was observed on the *gpsX *mutant or wild type strain inoculated leaves at 7 days post inoculation (dpi); however, the *gpsX *mutant induced less canker lesions than the wild-type strain 306 at 14 dpi (Figure 4A). When infiltrated at a higher concentration (10^8 ^cfu/ml), the *gpsX *mutant induced significantly less lesions than wild type at 7 dpi, but caused similar disease symptoms as wild type at 14 dpi. In both cases, the complemented mutant strain with the intact *gpsX *cloned in pUFR053 showed no difference from the wild type strain (Figure 4A). Plant inoculation by spray, a method that mimics the natural infection, showed that the *gpsX *mutant was reduced in virulence on grapefruit compared with the wild-type strain 306. After 21 days post inoculation the number of canker lesions on leaves infected with the *gpsX *mutant was significantly less than that inoculated with wild type strain. Symptom induction by the *gpsX *mutant could be restored to the wild-type level by complementary plasmid pJU3110, but not by the empty vector (Figure 4B).

**Figure 5:**
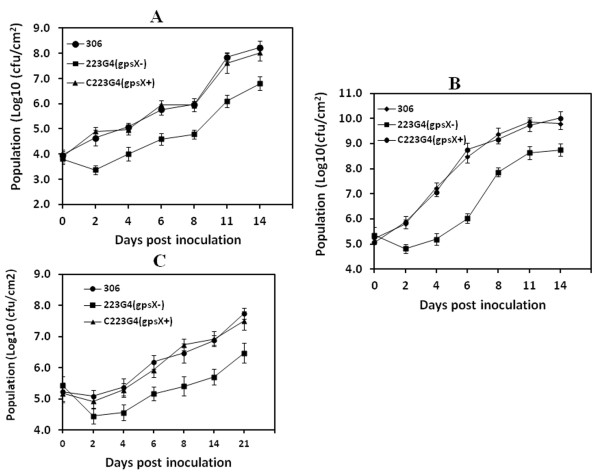
**GpsX is important for growth *in planta *of *X. citri *subsp. *citri***. (A) Growth of wild-type strain 306 and its derivatives in inoculated grapefruit leaves by pressure infiltration with a concentration at 10^5 ^cfu/ml. 306: wild-type strain 306; 223 G4(gpsX-): *gpsX *mutant; C223G4 (gpsX*+*): complemented *gpsX *mutant. (B) Growth of wild-type strain 306 and its derivatives in inoculated grapefruit leaves by pressure infiltration with a concentration at 10^8 ^cfu/ml. (C) Growth of wild-type strain 306 and its derivatives in inoculated grapefruit leaves by spray with a concentration at 10^8 ^cfu/ml. Bacterial cells were extracted from the leaves at different time points after inoculation, plated on appropriate media after serial dilution, and colonies counted after a 2-day incubation at 28°C. The values shown are means of three repeats and standard deviations. All the assays were repeated three times with similar results.

Although there were no differences between the wild type and the *gpsX *mutant strains in the ability to grow in XVM2 medium (data not shown), the growth of *gpsX *mutant 223 G4 (*gpsX*-) was significantly reduced *in planta *compared to the growth of the wild-type strain. After inoculation by infiltration at 10^5 ^cfu/ml, the bacterial population of the *gpsX *mutant moderately reduced *in planta*, and between 24 and 48 h, the bacterial population began to increase; whereas the bacterial population of the wild type strain 306 continued to increase after inoculation (Figure 5A). The bacterial population of the *gpsX *mutant recovered from the infected leaves was approximately 10 to 100-fold lower than that of the wild-type strain at each of the test points (Figure 5A). Similar differences in growth of the wild type and mutant strains were observed following infiltration at 10^8 ^cfu/ml (Figure 5B). The bacterial population of the complemented strain was similar to that of the wild-type at each test point (Figure 5A and 5B). The populations of the *gpsX *mutant compared to wild type strain were also analyzed after inoculation via spraying and significant differences were observed. As shown in Figure 5C, after inoculation, the population of the wild type strain remained approximately constant until 4 dpi, whereas the population of the *gpsX *mutant declined significantly. At 4 dpi, the population size of the *gpsX *mutant was nearly 100 times lower than for the wild type strain. From that point forward, the population sizes of the *gpsX *mutant began to increase slowly, whereas growth of the wild type strain continued after inoculation, so that, at 14 dpi, the difference in population size was one to two orders of magnitude. The affected growth of the *gpsX *mutant was restored to wild type levels by complementation with the cloned *gpsX *gene (Figure 5C). Overall, these findings suggest that *gpsX *is required for *X. citri *subsp. *citri *to proliferate well and to achieve full virulence in host plants.

### Mutation in *gpsX *affected biofilm formation of *X. citri *subsp. *citri *on abiotic surfaces and host leaves

**Figure 6:**
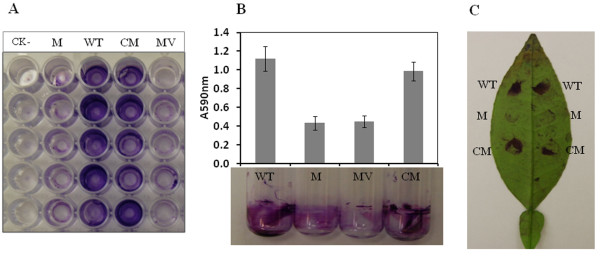
**Biofilm formation by *X*. *citri *subsp. *citri *strain 306 and its derivatives**. Biofilm formation in polystyrene 96-well plates (A), glass tubes (B) and on citrus abaxial leaf surfaces (C) was visualized using crystal violet staining. Biofilm formations in glass tubes were quantified by measuring the optical density at 590 nm after dissolution in ethanol-acetone (80:20, v/v). WT: wild-type strain 306; M: *gpsX *mutant 223 G4 (*gpsX-*); MV: *gpsX *mutant 223G4V (*gpsX-*) with empty vector pUFR053; CM: complemented *gpsX *mutant C223G4 (*gpsX+*); CK-: XVM2 medium without inoculation of bacteria. All experiments were performed in quadruplicate and repeated three times with similar results, and only one representative result is presented. Means ± standard deviations are shown.

Biofilm has been well characterized as a virulence trait in many plant pathogenic bacteria [36]. Our earlier study indicated that *gpsX *is related to biofilm formation [24]. In order to confirm the role of *gpsX *in biofilm formation in *X. citri *subsp. *citri*, biofilm formation of the *gpsX *mutant was examined on three different kinds of surfaces: polystyrene, glass and host leaves. The *gpsX *mutant 223 G4 (*gpsX-) *exhibited a significant reduction in biofilm formation both on polystyrene surface and in glass tubes compared to that of the wild-type, where the level of biofilm formation were approximately 30% and 40% of the wild-type level, respectively; and the complemented C223G4 (*gpsX+) *strains were restored to levels similar to that of the wild-type strain (Figure 6A and 6B). Similar to the observations on polystyrene surface and in glass tubes, the *gpsX *mutant showed declined biofilm formation on citrus leaf surfaces and, the complemented C223G4 (*gpsX+) *strains were restored the wild-type levels (Figure 6C), suggesting that the *gpsX *gene is involved in biofilm formation of *X*. *citri *subsp. *citri *cells on citrus leaves. These findings confirmed that the *gpsX *gene is involved in biofilm formation in *X*. *citri *subsp. *citri*.

### Mutation of *gpsX *caused impairment in cell motility but no impact on flagellar formation

**Figure 7:**
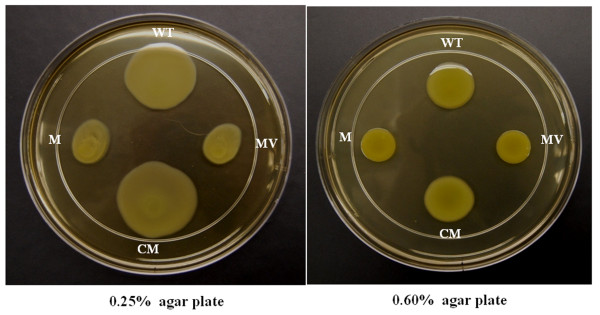
**Motilities of *X*. *citri *subsp. *citri *strains**. Cells were inoculated onto NA plates supplemented with 0.25% or 0.60% agar from bacterial cultures at exponential stage and photographed after 3 days (0.25% agar plate) and 7 days (0.60% agar plate) of incubation at room temperature (22-23°C). WT: wild-type strain 306; M: *gpsX *mutant 223 G4 (*gpsX-*); MV: *gpsX *mutant 223G4V (*gpsX-*) with empty vector pUFR053; CM: complemented *gpsX *mutant C223G4 (*gpsX+*). The *gpsX *mutant 223 G4 (*gpsX-*) is reduced in cell motility, which could be restored to the wild-type level by providing a plasmid bearing the *gpsX *gene [i.e., the complemented strain C223G4 (*gpsX+*)]

Several studies have indicated that both EPS and LPS are associated with the flagella-independent motility in a couple of bacteria including *X*. *citri *subsp. *citri *[21,24,37]. To verify whether a mutation in *gpsX *has any effect on the cell motility of *X*. *citri *subsp. *citri*, the *gpsX *mutant was evaluated for the mobile ability on swimming and swarming plates, respectively. The results showed that a significant reduction (*P *< 0.05, student's t-test) both in swimming and swarming motility was observed in the *gpsX *mutant 223 G4 (*gpsX-)*, compared with the wild-type strain (Figure 7). On the tested plates, the diameter of the growth zones resulting from migration away from the inoculation points on the agar surface were about 2.5 cm (swimming plates) and 2.0 cm (swarming plates) for the *gpsX *mutant, and 4.2 cm (swimming plates) and 3.0 cm (swarming plates) for the wild type. The diameter of the complemented strain and the wild-type strain were not significantly different, indicating that the mobility of the mutant could be restored to wild-type levels by *gpsX *in *trans*. Flagellum visualization assays using transmission electron microscope (TEM) showed that both the wild-type and the *gpsX *mutant strains formed a polar flagellum at the cell surface (data not shown), suggesting that mutation of *gpsX *has no impact on flagellar formation in *Xac*. These results indicated that the *gpsX *is implicated in bacterial motility in *X*. *citri *subsp. *citri*.

### GpsX contributed to stress tolerance of *X. citri *subsp. *citri*

The decrease in bacterial population *in planta *of the *gpsX *mutant immediately after inoculation (Figure 5A, B and 5C) suggested that the *gpsX *gene might play a role in the adaptation of *X*. *citri *subsp. *citri *to the conditions of the host microenvironments. To test this hypothesis, the survival of the *gpsX *mutant was investigated under various stresses that would be likely experienced at the early stage of infection when the bacteria has to attach to the leaf surface and later when the bacteria has to survive inside the host plant, including UV radiation, heat shock, saline stress, osmotic challenge, desiccation stress, SDS exposure and the H_2_O_2 _oxidative stress. These assays revealed that the *gpsX *mutant 223 G4 (*gpsX-*) was more sensitive than the wild-type strain to UV radiation, heat shock, desiccation stress, SDS exposure, and H_2_O_2 _(Table 4). After 20 min of exposure to UV radiation, there were greater numbers of surviving cells of the wild-type strain than that of the *gpsX *mutant. Following 15 min of exposure of bacteria to heat (50°C), viable cells of the *gpsX *mutant declined more rapidly than the wild-type. When exposed to air and dried for 60 min, the *gpsX *mutant showed significantly decreased survival compared with the wild-type strain. After treatment with SDS (0.1%) for 10 min, the survival rate of the *gpsX *mutant was significantly lower than that of the wild-type strain. The *gpsX *mutant also showed higher sensitivity than the wild type strain to hydrogen peroxide (exposure to 0.03% H_2_O_2 _for 20 min). The levels of stress tolerance of the complemented strain were similar to those of the wild type (Table 4), indicating that the affected stress tolerance of the *gpsX *mutant could be restored by *gpsX in trans*. There were no differences between the *gpsX *mutant and wild type strain in survival under saline stress or osmotic challenge.

**Table 4:** Survival of the *gpsX *mutant and wild-type *X.citri *subsp. *citri strain *306 under multiple stresses^A^

Strains	Survival rate (%)^B^						
	
	UV radiation	Heat shock	Desiccation tolerance	SDS exposure	H_2_O_2 _exposure	Osmolarity stress	Saline stress
306	3.2 ± 1.2a	0.04 ± 0.02a	2.7 ± 0.7a	10.1 ± 3.1a	1.6 ± 0.5a	4.9 ± 2.3a	6.1 ± 2.4 a
223G4 (*gpsX-*)	0.9 ± 0.3b	0.004 ± 0.003b	0.4 ± 0.1b	0.05 ± 0.02 b	0.05 ± 0.02b	3.8 ± 1.4a	3.9 ± 2.2 a
223G4V (*gpsX-*)	1.1 ± 0.5b	0.005 ± 0.003b	0.7 ± 0.2b	0.08 ± 0.03 b	0.12 ± 0.04b	4.1 ± 1.7a	5.5 ± 1.7 a
C223G4 (*gpsX+*)	4.2 ± 1.6a	0.05 ± 0.03a	3.5 ± 1.3a	8.2 ± 2.5a	2.2 ± 0.4a	5.5 ± 2.4a	7.4 ± 2.8 a

^A^Bacterial cell viability was estimated by plating on NA agar before (T0) and after (T1) the treatment. Percentage survival was calculated as the ratio of viable cell counts at T1 to that at T0. The treatments were applied as follows: for UV radiation, overnight bacterial culture was exposed to short-wave UV irradiation (254 nm) for 20 min; for heat-shock, bacterial culture was incubated at 50°C for 15 min; for desiccation stress, bacterial culture was placed on glass coverslips and air dried in a laminar flow apparatus for 60 min and then resuspended in 0.85% NaCl and plated; for SDS exposure, bacterial culture was treated with 0.1% SDS for 20 min; for sensitivity to hydrogen peroxide, bacterial culture was exposed to 0.03% H_2_O_2 _for 20 min; for osmotic stress, bacterial culture was treated with 40% D-sorbitol for 40 min; for saline stress, bacterial culture was treated with 1.0 M NaCl for 20 min. Bacterial cells were serially diluted with NB medium and colony-forming units (cfu) were counted after being cultured on NA plates at 28°C for 48 h. Each test, plated in triplicate, was repeated three times with similar results. ^B ^Data shown are means and standard errors of three replicates from one representative experiment. Different letters in each data column indicate significant differences at *P *< 0.05 (Student's t-test).

### Mutation of *gpsX *has no impact on expression of virulence-related genes

Reduced virulence could result from down-regulation of key virulence genes. In order to test whether mutation of the *gpsX *gene affected the expression of virulence-related genes, quantitative reverse transcription-PCR (QRT-PCR) assays were performed to monitor the expression profiles of six genes which were selected based on the alternated mutant phenotypes mentioned above. For total RNA preparation, the *gpsX *mutant and wild type strains were cultured to exponential phase in XVM2 medium that has been reported to mimic the environment of plant intercellular spaces [38]. The six target genes included one EPS biosynthesis gene (*gumB*), one LPS synthesis gene (*rfbC*), one catalase gene (*katE)*, one TTSS component gene (*hrcV*), one TTSS regulator genes *hrpX*, and one TTSS effector gene (*pthA*). The results showed that none of the six genes was significantly differently expressed in the mutant 223 G4 (*gpsX-*) compared with wild-type strains when grown in XVM2 medium (Table 5), based on a student's t-test (*P *< 0.05). Specifically, the primer set used for *pthA *is present in *pthA4 *and its homologues *pthA1*, *pthA2*, and *pthA3*, but not in any other genes. Thus we refer it as *pthA *rather than differentiating them. The qRT-PCR result based on this primer should detect the expression of *pthA4*, *pthA1*, *pthA2*, and *pthA3*. It is very likely that *pthA4*, *pthA1*, *pthA2*, and *pthA3 *have similar gene expression pattern due to the same promoter sequences. The sequences are 100% identical in the 213 bp upstream of *pthA4*, *pthA1*, *pthA2*, and *pthA3 *including the predicated promoter region (data not shown). Consequently, the qRT-PCR result will represent the relative fold change in gene expression for *pthA4*, *pthA1*, *pthA2*, and/or *pthA3 *since it is relative fold change and not absolute expression value.

**Table 5:** Comparison of virulence gene expression in the wild type and the *gpsX *mutant cultured in XVM2 medium by QRT-PCR *^a^*

Gene	locus_tag	Function of protein product	ΔΔ*C_T _*± SD*^b^*	Fold change ± SD *^c^*	*P^d^*
*gumB*	*XAC2585*	EPS xanthan biosynthesis	0.2665 ± 0.1912	0.8314 ± 0.1102	0.0524
*rfbC*	*XAC3598*	LPS O-antigen biosynthesis	-0.2018 ± 0.1467	0.8695 ± 0.0841	0.0621
*katE*	*XAC1211*	Monofunctional catalase	0.0758 ± 0.1346	0.9485 ± 0.0871	0.4407
*pthA*	NS *^e^*	TTSS effector	-0.1703 ± 0.2407	1.1253 ± 0.1845	0.3128
*hrpX*	*XAC1266*	TTSS regulator	0.2578 ± 0.1638	0.8364 ± 0.0997	0.2442
*hrcV*	*XAC0405*	TTSS component	0.1828 ± 0.1348	0.8811 ± 0.0832	0.1119

*^a ^*Both 16S rRNA and *gyrA *genes were used as endogenous controls in the QRT-PCR experiments and similar results were obtained when the data were normalized against the two genes respectively. Only the data obtained with 16S rRNA gene as control were shown.

*^b ^*The mean *ΔΔC_T _*was determined using four biological repeats. The experiment was repeated two times with similar results. Data from one experiment are shown.

*^c ^*The expression change (mutant/wild type) in mutant 223 G4 (*gpsX-*) was calculated using 2^-*ΔΔCT*^.

*^d^P *value, analyzed by Student's *t -*test. Values are significantly different when *P *is < 0.05.

*^e ^*No specific locus_tag. This represents the gene expression of *pthA1*, *pthA2*, *pthA3 *and *pthA4*.

## Discussion

In this work we have extended the characterization of the XAC3110 gene locus, previously identified and named *bdp24 *for involvement in *Xac *biofilm formation [24]. We conclude from several independent lines of evidence that this gene is required for EPS and LPS biosynthesis, and consequently required for biofilm formation and full virulence of *Xac *on host plants. For this reason, we have changed the name of this gene to *gpsX*, for glycosyltransferase for polysaccharide synthesis in *Xac*, to reflect the apparent multiple function of the gene product.

Several lines of evidence indicate that the *gpsX *locus is involved in polysaccharide biosynthesis. First, GpsX contains a glycosyltransferase family 2 domain and shares the conserved catalytic residues of glycosyltransferases (Figure 1 and 2). Second, mutation of *gpsX *resulted in decreased production of EPS (Figure 3A, Table 3) and altered LPS synthesis (Figure 3B), consistent with the general role of glycosyltransferases in polysaccharide biosynthesis [12,13]. Third, similar genes associated with polysaccharide biosynthesis have been identified in other bacterial pathogens (see below). Homologues of GpsX widely occur in the genomes of related phytopathogenic bacteria of *Xanthomonas *(Table 1). The biochemical characteristics and physiological roles of these homologous proteins remain unknown.

Some glycosyltransferase genes have already been identified in *Xanthomonas *spp. For example, as mentioned previously, the *rfbC *gene encodes a glycosyltransferase, which serves as a truncated O-antigen biosynthesis protein involved in LPS production in *X. citri *subsp. *citri *[23]. Both the ORFs *XC_3814 *and *XC_3555 (xagB) *in *X. campestris *pv. *campestris *are implicated in EPS production, but not LPS production [21,22]. In addition, the *gumDMHKI *genes encode different glycosyltransferases that direct the biosynthesis of the xanthan EPS in *X. campestris *pv. *campestris *[39,40]. However, these enzymes in *Xanthomonas *are mono-functional, i.e., involved either in EPS or LPS production. Our data showed that the *gpsX *gene is involved in both EPS and LPS production (Figure 3). The low similarities between GpsX and these proteins (data not shown) may suggest the differences in substrates and products.

Bacterial polysaccharides are usually synthesized from intracellular nucleotide sugar precursors and, most bacterial polysaccharides contain polymerized saccharide repeating units, the assembly of which involves glycosyltransferases that sequentially link monosaccharide moieties from nucleotide sugars to the growing sugar chain (saccharide acceptors) [11]. Different classes of bacterial polysaccharides can be distinguished on basis of their biosynthesis mechanisms and the precursors required. However, it is worth mentioning that, in some instances, mutation of single genes simultaneously affected biosynthesis of different polysaccharides, similar with the observation in this work. For example, in *X. campestris *pv. *citrumelo*, the mutation in *opsX*, a homologue of *waaF *(*rfaF*) which codes for a heptosyltransferase for LPS synthesis in *E. coli*, affected biosynthesis of LPS and EPS [41]. In addition, mutants in *xanA *and *xanB*, involved in UDP-Glucose and GDP-Mannose biosynthesis in *X. campestris *pv. *campestris*, respectively, showed a decrease in EPS production and an altered LPS [42]. Mutants in *galE*, encoding a UDP-galactose epimerase in *Erwinia amylovora*, were deficient in EPS production and produced a LPS with an altered side chain structure [43]. The dual effect of certain genes on EPS and LPS may be due to the shared pathways for EPS and LPS synthesis in these bacteria. As discovered in *Salmonella*, the same precursor molecule, UDP-glucose, is used for LPS O-antigen polysaccharide and capsular polysaccharide [44]. The major EPS produced by xanthomonads, xanthan, composed of polymerized pentasaccharide repeating units, consisting of glucose, mannose and glucuronic acid [39]. Most recently, glucose and mannose were found to be components of LPS in *X. citri *subsp. *citri *[45]. Given the altered O-antigen containing LPS profile of the *gpsX *mutant and its decreased level of EPS production, it was likely that the *gpsX*-encoded glycosyltransferase was involved in the formation of saccharide repeating units that might be found in *X. citri *subsp. *citri *EPS and LPS, by transferring the glucose and/or mannose monosaccharide moiety from certain nucleotide sugar precursors to corresponding acceptors. However, biochemical evidence for this proposed function of GpsX is needed.

Interestingly, the *gpsX *gene is located outside of the LPS gene cluster even though it is involved in the O-antigen biosynthesis. The LPS cluster is responsible for synthesis of O-antigen polysaccharide. The locus presents a size of 19.9 kb and contains 17 ORF [7,46]. The LPS cluster contains three glycosyltransferases, i.e. XAC3598 (RfbC), ORF5, and XAC3595. RfbC was annotated as a 614-amino-acid truncated O-antigen biosynthesis protein containing two separate glycosyltransferase family 2 (GT2) domains. The involvement of *rfbC *in O-antigen biosynthesis has been confirmed in our previous study [23]. The *orf5 *has been annotated to encode a putative glycosyltransferase [46], whereas XAC3595 shows significant homology to the glycosyltransferase A (GtrA) family [46]. It remains to be determined how GpsX and other glycosyltransferases are involved in O-antigen biosynthesis in Xac.

The attenuation in virulence and growth *in planta *of the *gpsX *mutant both in epiphytic (Spray) and wound (pressure infiltration) inoculations (Figure 4 and 5) may result, at least partially if not completely, from the reduction in EPS production (Figure 3A) and the alteration of LPS profile (Figure 3B), and consequently impaired cell motility (Figure 7) and biofilm formation (Figure 6), rather than from an effect on the virulence genes (Table 5). EPS has been shown to act as an important virulence factor that contributes to epiphytic survival and/or bacterial *in planta *growth and disease symptom formation in several *Xanthomonas *spp. including *X. campestris *pv. *campestris*, *X. oryzae *pv. *oryzae*, and *X. citri *subsp. *citri *[8]. EPS can suppress plant basal defense responses by chelating divalent calcium that are require for the activation of plant defense responses [47,48]. It also contributes to biofilm formation [21,24,34,49], which promotes bacterial resistance to environment stresses [23,36]. LPS has also been shown to be an important virulence factor in various plant pathogenic bacteria including several *Xanthomonas *spp. [8], *Erwinia amylovora *[50] and *Pseudomonas syringae *[51]. It can serve as a physical barrier protecting bacteria from plant defense responses [51]. It may also contribute to biofilm formation [23,24]. In addition, both EPS and LPS are related to cell motility in a couple of bacteria including *X*. *citri *subsp. *citri *[21,24,37]. In certain phytopathogenic bacteria, e.g., *E. amylovora*, *P. syringae*, and *Ralstonia solanacearum*, motility has been suggested to contribute to bacterial virulence in the early stages such as invasion and colonization [52-54]. *X. citri *subsp. *citri *is an intercellular space-colonizing pathogen that invades host plants via stomata or wounds, and multiplies in the apoplasts [4]. Before entering the host, the pathogen persists as epiphytes on the plant surface and has to confront environment stresses. Once entering the host, the pathogen needs to tolerate preformed defense molecules to establish a successful infection. Therefore, the alteration of LPS of the *gpsX *mutant and the reduction in its ability to produce EPS and to form biofilm formation may attenuate its adaptation to the conditions of the host microenvironment, which result in its growth deficiency, and consequently reduced virulence. Consistent with this, it has been demonstrated that both EPS and LPS biosyntheses are required for growth and survival on leaf surfaces and full virulence in *X. citri *subsp. *citri *[23,34]. Finally, *gpsX *may aid bacterial survival at early stage of infection when the bacterium attaches to the leaf surface and later survives inside the plant tissue. Consistent with the hypothesis, the *gpsX *mutant was attenuated in resistance against various stresses including oxidative stress (Table 4), which is one of the early plant defense responses triggered by bacterial infections [55].

In summary, in this work we expanded the knowledge about the function of the novel glycosyltransferase encoding gene *gpsX *from *X. citri *subsp. *citri*. Based on its apparently unique function in polysaccharide synthesis and the widely conserved occurrence in sequenced strains of *Xanthomonas*, this enzyme may represent a novel virulence-related factor of phytopathogenic *Xanthomonas *including *X. citri *subsp. *citri*. Additional study of this gene and its protein product should yield new insights into the biochemistry and physiological roles of bacterial glycosyltransferase of the citrus canker bacterium *X. citri *subsp. *citri*.

## Conclusions

In this report we characterized the novel *gpsX *gene in *X. citri *subsp. *citri*. We demonstrated that the *gpsX *mutant is affected in EPS and LPS production, cell motility, biofilm formation, stress tolerance, growth *in planta*, and virulence on host plants and that the genetic complementation with the wild type *gpsX *gene, fully restored the affected phenotypes of the *gpsX *mutant to wild-type levels. In conclusion, the *gpsX *gene is important for polysaccharide synthesis and biofilm formation and thus, plays an important role in the adaptation of *X. citri *subsp. *citri *to the host microenvironments at early stage of infection and required for full virulence on host plants.

## Methods

### Bacterial strains, plasmids and growth conditions

The bacterial strains and plasmids used in this study are listed in Table 2. *E*. *coli *strains were grown in Luria-Bertani (LB) medium at 37°C. *Xac *wild type strian306 (rifamycin resistant) and the EZ-Tn5 insertion mutant strain 223 G4 (*gpsX-*) have been described previously [24]. *Xac *strains were grown in nutrient broth/agar (NB/NA) or XVM2 medium [38] at 28°C. Antibiotics were added at the following concentrations when required: ampicillin (Am) 50 μg/ml; chloramphenicol (Cm), 35 μg/ml; gentamycin (Gm), 5 μg/ml; Kanamycin (Km), 50 μg/ml; and rifamycin (Rf), 50 μg/ml.

### DNA manipulations

Bacterial genomic DNA and plasmid DNA were extracted using a Wizard genomic DNA purification kit and a Wizard miniprep DNA purification system following manufactuer's instructions (Promega, Madison, WI, USA). The concentration and purity of DNA were determined using a Nanodrop ND-1000 spectrophotometer (NanoDrop Technologies, Wilmington, DE, USA). The standard methods [56] were used for restriction digestion, DNA ligation, agarose gel electrophoresis and DNA transformation of *E. coli*. The restriction endonucleases, T4 DNA ligase and *Pfu *polymerase were provided by Promega (Promega Corporation, Madison, WI).

### Complementation of the *gpsX *mutant

For complementation of the *gpsX *mutant 223 G4 (*gpsX-*), a 2,299-bp DNA fragment containing the intact open reading frame (ORF) of *gpsX *and 230 bp upstream of the 5' end to 21 bp downstream of the 3' end of the ORF, was amplified from the genomic DNA of *Xac *strain 306 using the primers C10-F (5' -tcgaggtaccgttggtgtcgtcctcgaaat-3') and C10-R (5' - tcgtaagcttctcaccccgcaataaacaac-3'), respectively containing *Kpn*I and *Hin*dIII restriction enzyme sites (underlined). The PCR product was digested with *Kpn*I and *Hin*dIII and cloned into the complementary vector pUFR053 [33] to construct the recombinant plasmid pJU3110 (Table 2). The recombinant plasmid was transferred into the *gpsX *mutant 223 G4 (*gpsX-*) by triparental conjugation as described elsewhere [57], resulting in strain C233G4 (*gpsX+*) (Table 2).

### Quantitative determination of EPS production

To estimate EPS production, strains were cultured in 100 ml NB or XVM2 liquid medium containing 2% (wt/vol) various sugars (fructose, galactose, glucose, maltose, mannose, sucrose, and xylose) at 28°C with shaking at 200 rpm for 24 hours (in NB) or 48 hours (in XVM2). EPS was precipitated from the culture supernatant at different time point post inoculation with ethanol, dried, and weighed as described elsewhere [35].

### Lipopolysaccharides (LPS) analysis

Bacterial strains were cultured at 28°C in NB or XVM2 liquid medium with shaking (200 rpm). Five-milliliter samples of cultures at the exponential stage were collected and the LPS samples were isolated as described previously [23]. LPS was separated by SDS-PAGE and visualized using silver staining following the manufacturer's instructions (Bio-Rad Laboratories, Inc., Hercules, CA). Standard LPS from *Salmonella entenica *serovar Typhimurium was obtained from Sigma. The test was performed three times independently.

### Capsule assays

Bacterial capsules were stained using a capsule-staining kit (Eng Scientific Inc., Clifton, NJ, USA) following the manufacturer's instructions. The samples were photographed using a light microscope (Leica DMLB2; Leica Microsystems GmbH, Wetzlar, Germany) with a digital camera. The experiment was repeated three times.

### Biofilm formation assays

Biofilm formation on polystyrene and glass surfaces were examined as described previously [23] with modifications. The average of four replicates was used for quantitative measurement. Assays of biofilm formation on leaf surfaces were conducted as described previously [58] with modifications. Briefly, 20 μl of each bacterial suspension (10^8 ^cfu/ml) was incubated on the abaxial surface of citrus leaves, and the leaves were kept at 28°C in a humidified chamber. After 24 h of incubation, biofilm formation on the leaf surface was visualized using crystal violet staining. The experiments were repeated three times.

### Motility assays

To test cell motility, 2 μL of bacterial cultures at the exponential stage in NB (OD_600 _of 0.8) was spotted onto NA plates (diameter, 150 mm; each containing 50 mL of NA) containing 0.25% (wt/vol) agar (Difco, Franklin Lakes, NJ) for swimming motility testing or 0.6% (wt/vol) agar for swarming motility testing. Plates were incubated at room temperature for 7 days. The diameters of the areas occupied by the strains were measured, and the values were used to indicate the motility of *Xac *strains. The experiment was repeated three times with three replicates each time.

### Electron microscopy

For flagella visualization, cells grown on NA plates were harvested at 48 hours post inoculation (hpi) and suspended in 0.85% NaCl. One drop of cell suspension was placed onto a 400-mesh Formvar carbon-coated grid. Excess water was removed by blotting onto Whatman filter paper no. 1 (Whatman Inc, Piscataway, NJ, USA). One drop of 1% uranyl acetate solution was then added, and excess solution was removed. The grids were left at room temperature for 30 min. Samples were viewed with a Philips FEI Morgagni 268 transmission electron microscope (FEI Company, Eindhoven, Netherlands) operating at 80 kV.

### Stress tolerance assays

The assays were performed as described previously with modifications [23]. Bacterial culture at early exponential stage (OD_600nm _= 0.1) in NB were used to test survival under stresses: UV radiation, heat shock, saline stress, osmotic challenge, desiccation stress, SDS stress and oxidative stress. In each stress treatment, cell viability was determined by plate-counting of cfu. The survival rate was defined as the percentage of viable cell counts from the culture with stress treatment compared with those from the non-treated culture. The stress treatments were applied as follows: for UV radiation, the cells were exposed to short-wave UV radiation (254 nm in a biological safety cabinet) at a distance of 60 cm for 20 min; for heat-shock stress, the culture was transferred to 50°C for 15 min; for sodium stress, NaCl (pH 7.5) was added to the bacterial culture at a final concentration of 1.0 M, and survival was estimated after 20 min, respectively; for osmotic challenge, D-sorbitol (pH 7.0) was added to the bacterial culture at a final concentration of 40%, and survival was estimated after 40 min; for desiccation stress, the bacterial culture was placed on glass coverslips (18 mm × 18 mm), air dried in a laminar flow apparatus for 60 min and then resuspended in 0.85% NaCl and plated; for SDS stress, SDS (pH 7.5) was added to the bacterial culture at a final concentration of 0.1%, and survival was estimated after 10 min; for oxidative stress, H_2_O_2 _was added to the bacterial culture at a final concentration of 0.03%, and survival was estimated after 20 min. Each stress test was repeated three times with three replicates each time. Student's *t*-test was used to test the significance of the differences.

### Pathogenicity assays

Pathogenicity assays were performed as described previously [59]. Briefly, fully expanded, immature leaves of young (about 10-week-old) grapefruit (*Citrus paradise *cv. Duncan grapefruit) were prepared in a quarantine greenhouse at the Citrus Research and Education Center, Lake Alfred, FL. The *X. citri *subsp. *citri *strains were cultured for 2 days on NA plates at 28°C and were re-suspended in sterile tap water. A bacterial suspension (10^8 ^or 10^5 ^cfu/ml) was injected into the intercellular spaces of leaves with a needleless syringe; and a bacterial suspension (10^8 ^cfu/ml) was inoculated on the leaf abaxial surface by a spray method. All plant inoculations involved a minimum of three immature leaves at a similar developmental stage from each plant, and three plants were inoculated for each bacterial strain. All the tests were repeated three times independently.

### Bacterial growth assays *in planta*

For *in planta *growth assays, bacterial strains were inoculated onto leaves of grapefruit as described above. Leaf discs (0.8 cm in diameter) randomly selected from inoculated leaves were excised with a cork borer and then ground in 1 mL of 0.85% (w/v) NaCl. The suspension were serially diluted and plated on NA plates containing appropriate antibiotics. Bacterial colonies were counted after incubation at 28°C for 48 h and the number of cfu per square centimeter of leaf tissue was calculated. The *in planta *growth was measured in quadruplicate and the assays were repeated three times independently.

### RNA prepare and quantitative reverse transcription-PCR (QRT-PCR)

Total RNA of *X. citri *subsp. *citri *cells cultured in XVM2 medium at exponential phase (14 h after inoculation) was isolated using RNA protect bacterial reagent (Qiagen, Valencia, CA) and RNeasy Mini Kit (Qiagen, Valencia, CA) and contaminated genomic DNA was removed using a TURBO DNA-free kit (Ambion, Austin, TX), following the manufacturer's instructions. RNA purity and quality were assessed with a NanoDrop ND-1000 spectrophotometer (NanoDrop Technologies, Wilmington, DE).

A one-step QRT-PCR was performed with a 7500 fast real-time PCR system (Applied Biosystems, Foster City, CA) using a QuantiTect SYBR green RT-PCR kit (Qiagen, Valencia, CA) following the manufacturer's instructions. The gene specific primers used were previously designed [35,59], except the DNA gyrase subunit A encoding gene *gyrA *(FP: 5' -CGTCACGTTGATCCGTTTGT-3' ; RP: 5' -GCTTGCTTCGTCCACTCCCT-3'), based on the genome sequence of strain 306. Those primers targeted the gum gene *gumB*, LPS O-antigen biosynthesis related gene *rfbC*, TTSS genes *hrpX *and *hrcV*, a catalase gene *katE*, the virulence factor *pthA*. The 16S rRNA and *gyrA *genes were used as endogenous controls. The relative fold change in target gene expression was calculated by using the formula 2^-ΔΔ*CT *^[60]. QRT-PCR was repeated twice with four independent biological replicates each time.

## Authors' contributions

JL and NW conceived and designed the experiments, performed the experiments, analyzed the data and wrote the paper. All authors read and approved the final manuscript
